# Risk of prostatitis in patients with type 2 diabetes mellitus: An observational retrospective cohort study of canagliflozin versus other antihyperglycemic agents using propensity score matching

**DOI:** 10.1371/journal.pone.0341745

**Published:** 2026-02-02

**Authors:** Zhong Yuan, Carolyn H. Jeffcoat, Saberi Rana Ali, Anthony G. Sena, Martijn J. Schuemie, Patrick B. Ryan, Sergio A. Fonseca

**Affiliations:** 1 Johnson & Johnson, Horsham, Pennsylvania, United States of America; 2 Johnson & Johnson, Titusville, New Jersey, United States of America; University of Diyala College of Medicine, IRAQ

## Abstract

**Purpose:**

Prostatitis has been reported in patients with type 2 diabetes mellitus (T2DM) receiving antihyperglycemic agents (AHAs). This study was conducted to evaluate the risk of prostatitis with canagliflozin in response to a specific Health Authority query.

**Methods:**

This retrospective cohort study used data from adult male patients with T2DM who were new users of canagliflozin (target) or comparators (empagliflozin, dapagliflozin, sitagliptin, and liraglutide). Data were obtained from 8 global administrative claims databases, transformed to a Common Data Model for consistent analysis across databases. Pairwise comparisons were conducted using propensity scores to match canagliflozin users to users of each comparator at a 1:n ratio (maximum n = 100). Hazard ratios were estimated using a Cox proportional hazards model, conditioned on the matched set.

**Results:**

A total of 388,893 adult male patients with T2DM received canagliflozin across databases (mean age, 51.2–71.7 years) and were matched to 657,134 patients receiving empagliflozin, 340,539 receiving dapagliflozin, 819,047 receiving sitagliptin, and 278,684 receiving liraglutide. On-treatment incidence rates showed that prostatitis was uncommon in the canagliflozin cohort in nearly all databases (4.2–7.7 per 1000 person-years) and were similar to those of the comparator treatments. The exception was a higher crude incidence rate in the Merative MarketScan^®^ Medicare Supplemental Database (10.1–12.1 per 1000 person-years). Propensity score matching achieved good balance in all available covariates, and effect estimates were relatively close to a hazard ratio of 1.0, varying on both sides of the null effect. Minimum detectable relative risks were low in most databases, and meta-analytic estimates were near 1.0, with all upper bounds <1.50. No association (either increased or decreased risk) was found with canagliflozin versus other AHAs.

**Conclusions:**

This analysis found no evidence of a statistically significantly increased risk of prostatitis among adult male patients with T2DM receiving canagliflozin compared with the other AHAs evaluated in this study.

## Introduction

Infections, particularly genitourinary infections, such as urinary tract infections (UTIs; e.g., cystitis, pyelonephritis) and genital tract infections (e.g., prostatitis), are common in patients with type 2 diabetes mellitus [1–4]. The predisposition may be the result of several factors, including glucosuria (i.e., an increased presence of glucose in urine), which can promote bacterial growth, an increased adherence of bacteria to uroepithelial cells among patients with suboptimal glycemic control, and immune dysfunction associated with hyperglycemia [2–5]. Incomplete bladder emptying may also increase the risk of genitourinary tract infections in men; in the setting of diabetes, autonomic neuropathy can be present and asymptomatic in a substantial proportion of patients [6]. The UTIs associated with sodium-glucose cotransporter 2 inhibitors (SGLT2is) are typically mild to moderate in intensity and respond to standard treatment. Severe events or events associated with hospital admission were reported in 0.1% to 0.4% of patients in clinical trials; kidney infections and sepsis were “rare” [1]. Men with type 2 diabetes mellitus are at increased risk of developing prostatitis compared with nondiabetic men, which can result in pain, fever, dysuria, urinary retention, and incontinence [7,8].

The SGLT2is (e.g., canagliflozin, empagliflozin, dapagliflozin) are a class of oral antihyperglycemic agents (AHAs) that are commonly prescribed to improve glycemic control in patients with type 2 diabetes mellitus [9,10]. As a result of the mechanism of action of SGLT2is, there is increased urinary glucose excretion which may increase the risk of genitourinary infections [2,3]. Although genitourinary infections were commonly reported as adverse events in SGLT2i clinical trials, several case studies have reported instances of more serious genitourinary infections, such as prostatitis, urosepsis, and pyelonephritis, in patients with type 2 diabetes mellitus who were treated with SGLT2is; however, causality has not been comprehensively evaluated and this association is hypothesized to be secondary in nature [1,11–13]. There is mixed support from meta-analyses regarding the association between SGLT2is and the risk of genitourinary infections [13–15], and data are lacking on the risk of prostatitis (separate from UTIs or genitourinary infections as a whole), as well as the specific etiologies, particularly in relation to the use of SGLT2is.

The current study was conducted to evaluate the risk of prostatitis with canagliflozin in response to a Health Authority query. The study aimed to evaluate the incidence of prostatitis in adult male patients with type 2 diabetes mellitus receiving canagliflozin versus other AHAs using real-world clinical data.

## Methods

### Study design and patient population

**Fig 1:**
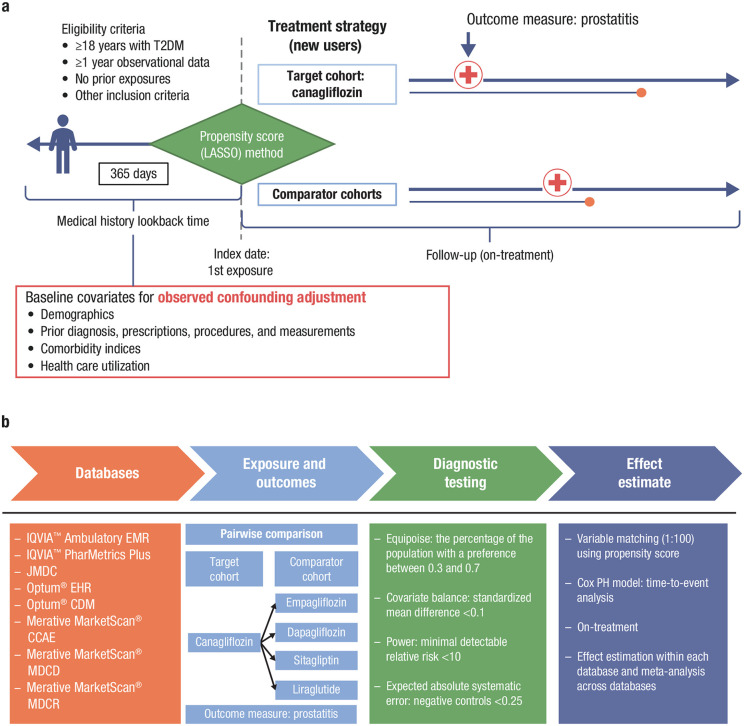
Comparative cohort (a) study design and (b) statistical analyses. CCAE, commercial claims and encounters; CDM, Clinformatics^®^ Data Mart; EHR, electronic health record; EMR, electronic medical record; LASSO, least absolute shrinkage and selection operator; MDC, medical data center; MDCD, multi-state Medicaid database; MDCR, Medicare supplemental; PH, proportional hazards; T2DM, type 2 diabetes mellitus.

This retrospective, observational, comparative, new-user cohort study evaluated data from adult men (aged ≥18 years) who were newly exposed to the target drug (canagliflozin) or comparator drugs (empagliflozin, dapagliflozin, sitagliptin, and liraglutide) during the study period lasting from 2013 to 2022 (with slight variation in dates of data availability across databases [ranges detailed in S1 to S5 Tables]). The comparator drugs were selected for several reasons: 1) to include a broad representation of different classes of AHAs (SGLT2is [empagliflozin and dapagliflozin], dipeptidyl peptidase-4 inhibitors [sitagliptin], and glucagon-like peptide 1 receptor agonists [liraglutide]); 2) they are highly effective agents commonly used in clinical practice for the management of type 2 diabetes mellitus; and 3) there is no well-established evidence linking these medications to the risk of prostatitis. High-level summaries of the overall study design are shown in Figs 1a and 1b, respectively.

Data were included for patients who had ≥ 1 diagnosis of type 2 diabetes mellitus, measurement of elevated hemoglobin A_1C_, or drug treatment for type 2 diabetes mellitus, with ≥1 type 2 diabetes mellitus diagnosis at any time prior to or within 365 days following the first treatment exposure (index date), and no diagnosis of type 1 diabetes mellitus or secondary diabetes. Diagnosis of type 2 diabetes mellitus was based on International Classification of Diseases (ICD)-9 and ICD-10 codes. Additionally, eligible patients had ≥ 365 days of observational data prior to the index date within the database to capture patient characteristics and establish a clean period. There were no other exclusion criteria.

For each treatment cohort, the at-risk period included all time exposed to the index drug, calculated as index date + 1 day until event occurrence, drug discontinuation, or the end of the observation period within the database. Drug discontinuation was determined by a 30-day period between the end of a given drug’s supply and any subsequent exposure (i.e., ≥ 60 days pass without refilling a 30-day prescription). Only the first at-risk period was considered for each patient within a given treatment cohort, though patients may have belonged to ≥1 cohort at different times.

### Data sources

Patient-level data from 8 global administrative claims databases were used for this study, including 7 United States (US) databases and 1 Japanese database. Data were accessed on December 14, 2022. The claims databases included: (1) IQVIA™ Ambulatory electronic medical records (IQVIA™ Ambulatory EMR), (2) IQVIA™ PharMetrics adjudicated health plan claims database (IQVIA™ PharMetrics Plus), (3) Optum’s de-identified electronic health record (Optum^®^ EHR), (4) Optum^®^ Clinformatics^®^ Data Mart – Extended Data Mart – Socioeconomic Status (Optum^®^ CDM), (5) Merative MarketScan^®^ Commercial Claims and Encounters Database (Merative MarketScan^®^ CCAE), (6) Merative MarketScan^®^ Multi-State Medicaid Database (Merative MarketScan^®^ MDCD), (7) Merative MarketScan^®^ Medicare Supplemental Database (Merative MarketScan^®^ MDCR), and (8) the Japan Medical Data Center claims database (JMDC). These databases are described in detail in S6 Table. All databases were transformed to the Observational Medical Outcomes Partnership Common Data Model, which provides a standardized representation of database structure and clinical content to enable consistent analysis across disparate health care databases [16,17]. The analyses used commercially licensed de-identified databases and no direct involvement with patients, so no one could identify the individual participants. All authors had access to information, including study reports and manuscript materials, which were aggregated information. The study analysts could have access to patient-level data.

### Outcome assessment

Cases were defined as having a prostatitis diagnosis (based on ICD-9 and ICD-10 codes, listed in S7 Table) following the index date, without the presence of testicular lesions, bladder neoplasm, or abdominal or inguinal hernia between 365 days before and 7 days after the index date. Events were identified as the earliest observed diagnosis of prostatitis and new events were defined after no evidence of history of or chronic prostatitis was observed in the previous 365 days. The case ascertainment method followed standard practices [18].

Negative control outcomes were used to estimate systematic error in each analytic design and enable empirical calibration [19]. Negative controls are outcomes believed not to be caused by any of the medications being studied. Any effect size estimates for negative control ideally should be close to the null. Negative control outcomes are defined as the first occurrence of the negative control concept or any of its descendants.

### Statistical analysis

The crude on-treatment incidence of prostatitis (per 1000 person years [PYs]) was estimated for each treatment cohort (i.e., canagliflozin, empagliflozin, dapagliflozin, sitagliptin, and liraglutide; Fig 1b). To control for confounding, a large-scale regularized logistic regression (least absolute shrinkage and selection operator [LASSO]) was used to fit propensity score (PS) model [20], and a variable ratio matching (up to 100 maximum) on PS was employed for each pairwise comparison between the canagliflozin cohort and the comparator treatment cohort [21]. The Large Scale Propensity Scores (LSPS) models were based on a large-scale regularized regression, which included a large, comprehensive set of baseline covariates. Prior research has suggested that under typical circumstances, LSPS may also potentially account for unmeasured confounding indirectly through measured covariates that correlate with unmeasured factors [22]. The covariates used for PS matching included medical history, demographics (i.e., age, sex, race/ethnicity, index year, index month), 30-day preindex variables (i.e., all conditions, all medication use, all procedures, all measurements), 365-day preindex variables (i.e., same as 30-day variables plus measurement results where available), overlapping index date (i.e., drug groups, which are categorized by Anatomical Therapeutic Chemical code, with index dates overlapping with canagliflozin or comparator AHAs), and comorbidity indices calculated using all prior medical history.

Prior to formal analyses, every combination of time-at-risk, comparator, and database (N = 32) was required to pass diagnostic testing that included standardized difference of covariates (mean difference, < 0.1), equipoise (percentage of population with a preference of 0.3–0.7) [23], minimum detectable relative risks at 80% power, and expected absolute systematic error (negative controls; also used for empirical calibration of results). Note that the preference score was calculated based on a transformation of the PS (ranging from 0 to 1) and it reflected the degree of overlap in treatment choices for different therapies involved in the study. Per the research by Walker et al [23], preference score values within the range of 0.3 to 0.7 suggest that there is a significant degree of similarity in the characteristics of patients receiving each of the treatments under comparison.

A conditional Cox proportional hazard model based on time-to-event analysis was used to estimate the hazard ratio (HR) within each individual database, conditioned on the matched set, with no adjustments made to account for multiple testing. The effect-size estimates across databases were combined using Bayesian random-effects meta-analysis based on nonnormal likelihood approximation to avoid bias due to small counts [24]. Empirical calibration was performed by first computing meta-analytic estimates for all negative controls, using those to fit empirical null distributions, and finally calibrating the meta-analytic estimates for the outcomes of interest.

### Ethical approval

Analyses of de-identified, publicly available data do not constitute human subjects research and, as such, do not require institutional review board review/approval.

## Results

### Study population

**Fig 2:**
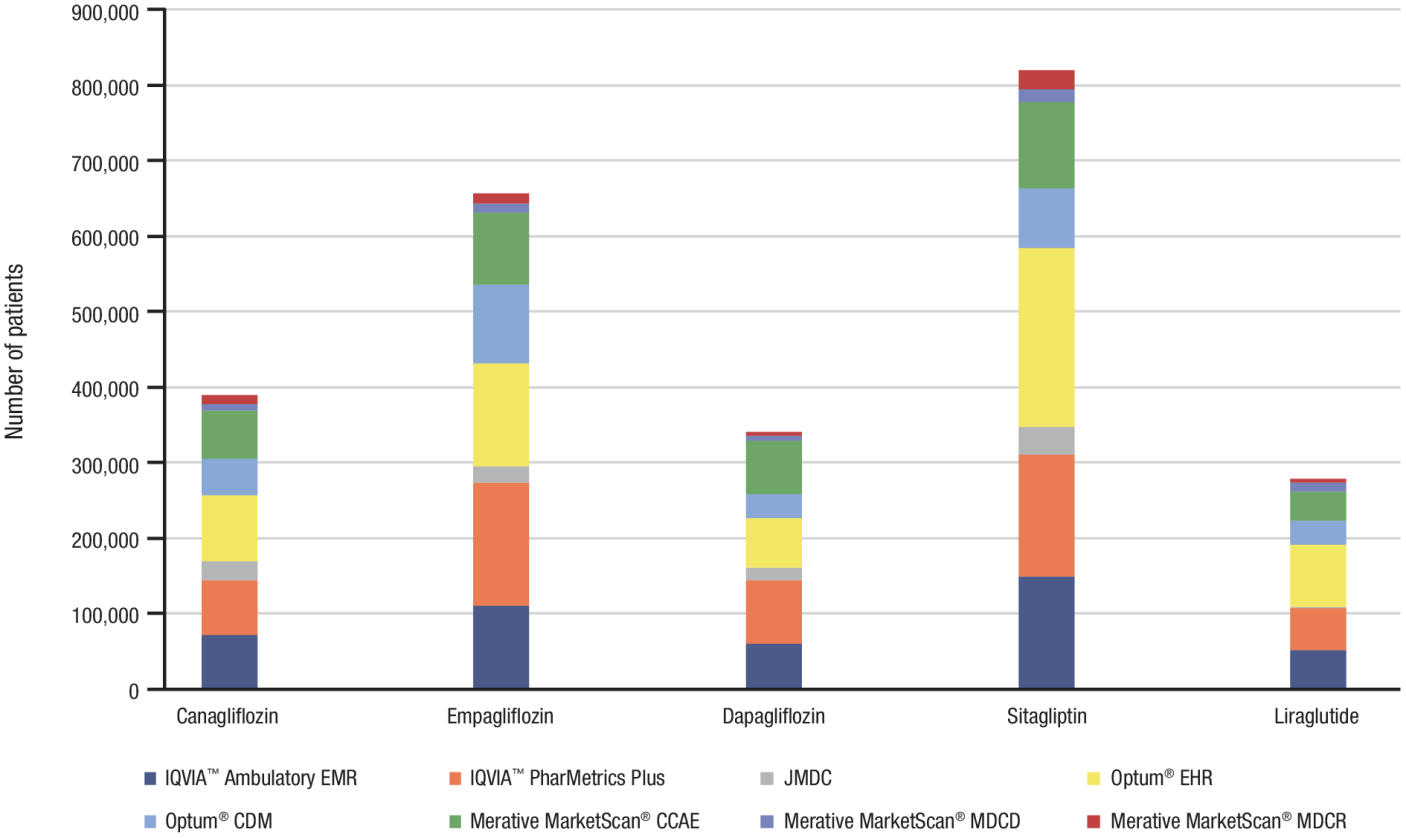
Sample size for each exposure cohort by database. CDM, Clinformatics^®^ Data Mart; CCAE, commercial claims and encounters; EHR, electronic health record; EMR, electronic medical record; MDC, medical data center; MDCD, multi-state Medicaid database; MDCR, Medicare supplemental.

Among the 8 databases that passed the diagnostic testing for inclusion in the statistical analyses comparison, the sample sizes were considerably larger for IQVIA™ Ambulatory EMR, IQVIA™ PharMetrics Plus, Optum^®^ EHR, Optum^®^ CDM, and Merative MarketScan^®^ CCAE compared with JMDC, Merative MarketScan^®^ MDCD, and Merative MarketScan^®^ MDCR (Fig 2). A total of 388,893 adult male patients with type 2 diabetes mellitus received canagliflozin, and were matched with 657,134 patients who received empagliflozin, 340,539 who received dapagliflozin, 819,047 who received sitagliptin, and 278,684 who received liraglutide. Across the databases, the mean age for new users of canagliflozin ranged from 51.2 (Merative MarketScan^®^ MDCD) to 71.7 years (Merative MarketScan^®^ MDCR). The baseline demographics and clinical characteristics of the 5 treatment cohorts are provided in the supporting information (S1 Table [canagliflozin] and S2 to S5 Tables [comparator AHAs]). The median treatment duration ranged from 4 to 6 months for most databases, except for IQVIA™ Ambulatory EMR and Optum^®^ EHR (approximately 1 month for each) and JMDC (approximately 10 months).

Baseline patient characteristics from the 8 databases were analyzed based on individual (not aggregated) verbatim terms. Across the databases, most patients in the canagliflozin cohort had hypertension/hypertensive disorder and hyperlipidemia; other common comorbidities included obesity, chest pain, low back pain, and respiratory illnesses during the baseline period (S1 Table). Other AHAs, antihypertensive medications, and cholesterol-lowering drugs were common concomitant treatments.

**Fig 3:**
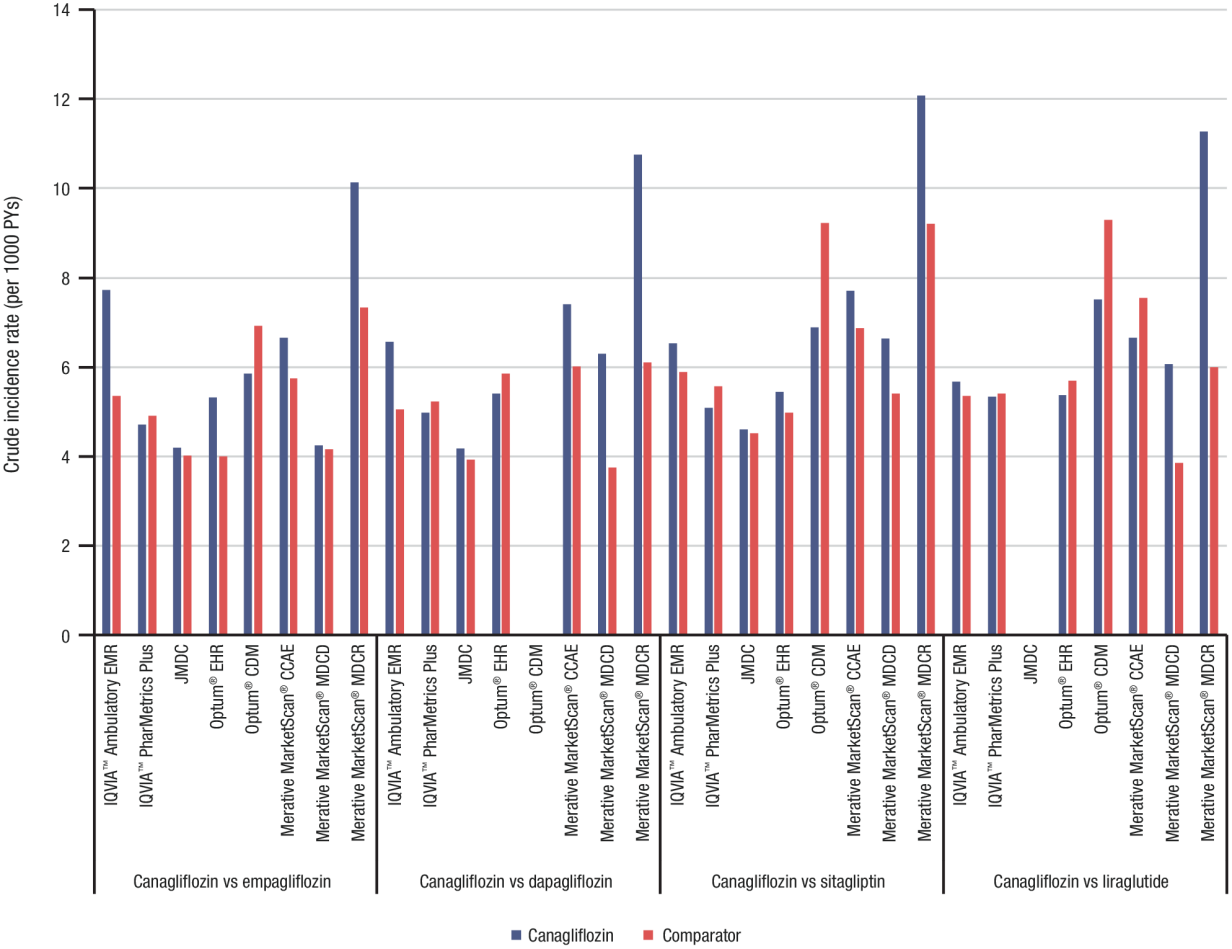
Crude on-treatment incidence rate (per 1000 PYs) of prostatitis for each treatment cohort by database. CDM, Clinformatics^®^ Data Mart; CCAE, commercial claims and encounters; EHR, electronic health record; EMR, electronic medical record; MDC, medical data center; MDCD, multi-state Medicaid database; MDCR, Medicare supplemental; PY, person-year.

During the follow-up period, the crude on-treatment incidence rates showed that prostatitis was uncommon among patients in the canagliflozin cohort in nearly all databases (range, 4.2–7.7 per 1000 PYs) and were similar to those observed for the 4 comparator cohorts. The exception to this was a higher crude incidence rate of prostatitis in the Merative MarketScan^®^ MDCR (range, 10.1–12.1 per 1000 PYs; Fig 3). The crude incidence rates and event counts are provided in S8 Table.

### Risk estimation

**Fig 4:**
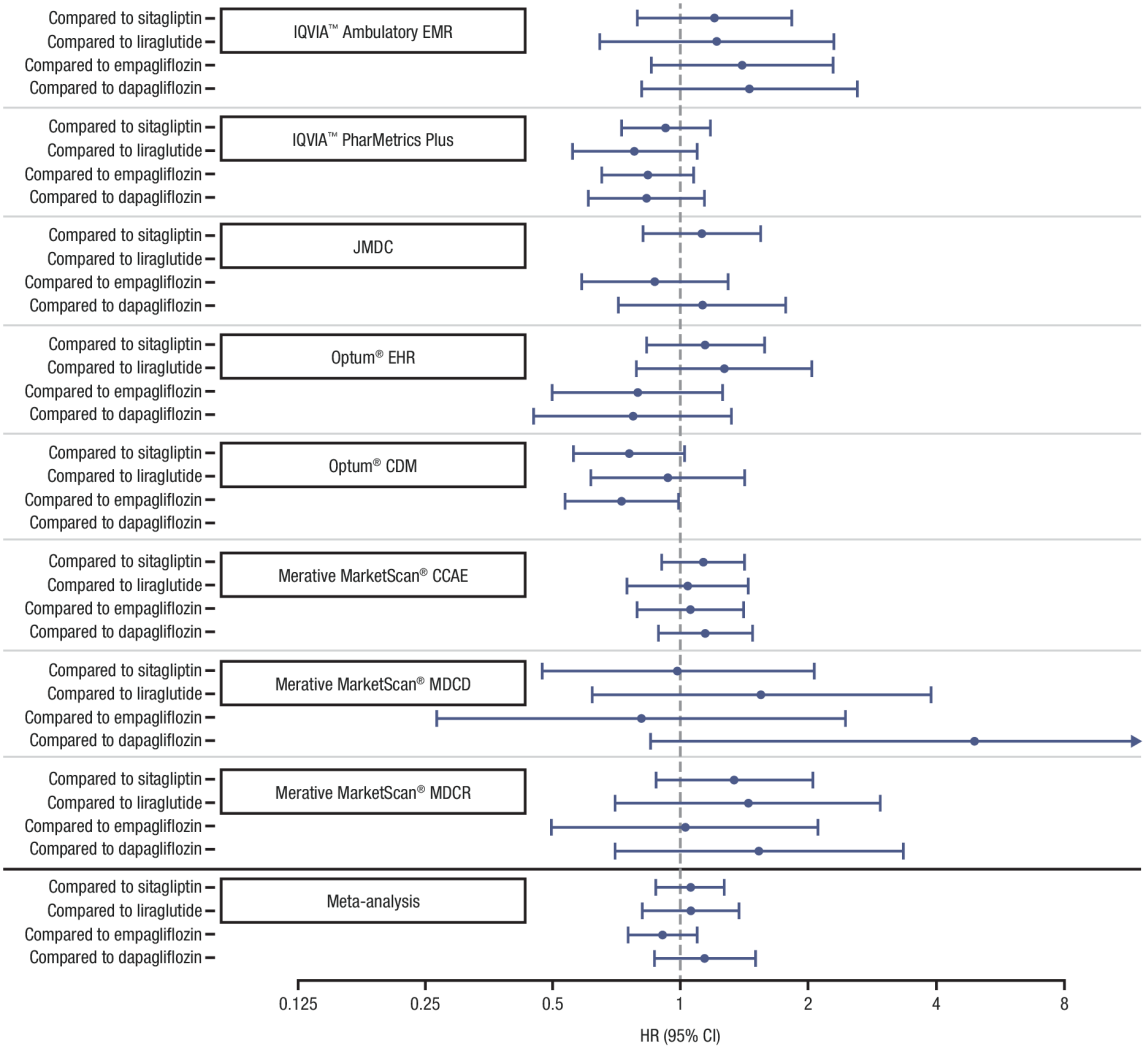
Effect estimation of pairwise comparison of on-treatment prostatitis syndrome events by database. CDM, Clinformatics^®^ Data Mart; CCAE, commercial claims and encounters; EHR, electronic health record; EMR, electronic medical record; MDC, medical data center; MDCD, multi-state Medicaid database; MDCR, Medicare supplemental.

After PS matching, all available covariates were well-matched between cohorts (as an example, covariate balance before and after PS matching using the IQVIA^TM^ Ambulatory EMR database is presented in S1 Fig). Effect estimates across the databases for prostatitis risk varied on both sides of the null effect, with the HR relatively close to 1.0. The minimum detectable relative risks were low in most databases, often <1.5, and almost always <2.0. Estimates from the meta-analysis were very close to 1.0 with upper bounds <1.50 for all pairwise comparisons (HR and 95% confidence interval [CI]): canagliflozin versus empagliflozin (0.91 [0.75–1.09]), canagliflozin versus dapagliflozin (1.14 [0.87–1.50]), canagliflozin versus sitagliptin (1.06 [0.88–1.27]), and canagliflozin versus liraglutide (1.06 [0.81–1.37]). There was neither a protective nor harmful association for risk of prostatitis with canagliflozin versus the comparator AHAs (i.e., empagliflozin, dapagliflozin, sitagliptin, and liraglutide; Fig 4).

## Discussion

Using real-world clinical data from 8 global claims databases that met diagnostic criteria, this retrospective, observational study compared the incidence of prostatitis in adult male patients with type 2 diabetes mellitus who were new users of canagliflozin to that of 4 comparator AHA treatments (i.e., empagliflozin, dapagliflozin, sitagliptin, and liraglutide). During the follow-up period, the crude on-treatment incidence rates showed that prostatitis was uncommon in the canagliflozin cohort in nearly all databases (range, 4.2–7.7 per 1000 PYs) and were similar to those found among comparator treatment cohorts. The exception to this observation was a higher crude incidence of prostatitis in 1 database (Merative MarketScan^®^ MDCR, range, 10.1–12.1 per 1000 PYs), which mainly consists of patients older than 65 years of age. After PS matching using all available covariates, there was no evidence of a statistically significantly increased risk of prostatitis in patients who received canagliflozin compared with the other AHAs evaluated in this study, including other SGLT2is. In a prior study using the Kaiser Permanente Northwest (Portland, Oregon) database, the incidence of physician-diagnosed prostatitis was 4.9 per 1000 PYs, although the study was not restricted to patients with type 2 diabetes mellitus [25].

While UTIs have been identified as adverse drug reactions associated with the use of SGLT2is, there is mixed support from other large data analyses for an association between SGLT2i use and an increased risk of genitourinary infections. Multiple meta-analyses have found SGLT2i use to be associated with an increased risk of genital mycotic infections but not UTIs [14,26], while a meta-analysis among older patients (≥65 years) found no increased incidence of genitourinary infections within 6 months of SGLT2i initiation [13]. It has been suggested that these disparities may be due to the specific SGLT2i molecule being studied, as initial meta-analyses were conducted using patient data from the earlier available drugs [27]. In more recent studies, the increased risk of UTI that was observed for dapagliflozin was not seen with either canagliflozin or empagliflozin [14,15,26,27]. Prostatitis has received limited attention in the literature separate from UTIs as a whole, but findings of the current study suggest that rates of prostatitis are low and similar in patients with type 2 diabetes mellitus who received treatment with the SGLT2i canagliflozin or 1 of the comparator drugs, with no statistically significantly increased risk of prostatitis from canagliflozin treatment versus any of the comparator drugs evaluated in this study.

The strengths of the current analysis include its use of well-established LSPS matching methods to control confounding. The results were robust, including very large patient cohorts and evaluating treatment consistency based on 9 years of data from routine clinical practice from 8 large global administrative claims databases. Analyses were required to pass stringent prespecified diagnostic testing and negative control outcomes were also implemented to evaluate any potential systematic study design bias and used for empirical calibration of the overall treatment effect estimates. Additionally, this analysis specifically evaluated the risk for prostatitis among patients with type 2 diabetes mellitus, whereas prior analyses have focused on risk assessments across broader classifications of infection, such as overall genitourinary infections.

However, this analysis has several limitations. Although the results showed no statistically significant association of prostatitis with canagliflozin over comparator AHAs, the observational design precludes completely ruling out a causal effect. While the Bayesian meta-analytical estimates for the risk of prostatitis with canagliflozin versus comparator AHAs showed point estimates very close to 1.0, a moderate risk increase cannot be ruled out as the upper bounds of the 95% CI were <1.50. The study used observational retrospective data that were originally collected for insurance claims purposes and medical care processing from routine clinical practice. Accordingly, data accuracy and the level of clinical details may be different than those from a data source that has been prospectively collected or specifically designed to address certain clinical questions. Data-related limitations include dependency on the accuracy of codes and algorithms to identify at risk conditions and the lack of details regarding the circumstances related to prostatitis diagnosis (e.g., urinalysis and urine culture results, prostate-specific variables like prostate-specific antigen levels or imaging results, and alternative etiologies). The use of a 1-year baseline period to capture covariates may not have accounted for long-term chronic conditions that could predispose patients to prostatitis. As the study includes data spanning from 2013 to 2022, the potential for temporal bias (changes in clinical guidelines, prescribing habits, or diagnostic criteria over time) cannot be excluded; this is an inherent limitation of real-world studies. The median follow-up period of 4–6 months for most databases is also relatively short for detecting prostatitis. Misclassification of participants with type 2 diabetes mellitus or prostatitis cannot be ruled out due to the use of ICD codes for diagnosis of these conditions. Bias due to non-random censoring is also a limitation of our study as treatment exposure was determined based on pharmacy drug dispensing records rather than actual administration. In addition, treatment discontinuation due to UTIs (a known SGLT2i risk) might have censored prostatitis events (if the prostatitis event proceeded shortly after a UTI event), potentially biasing the HRs for the risk of prostatitis. The study findings may also be subject to residual confounding effects due to its observational nature and unmeasured confounding such as the frequency of prostate examinations, antibiotic use, sexual history, and lifestyle factors (e.g., smoking, alcohol consumption). Therefore, it was critically essential that our analysis used LSPS methods to minimize the residual confounding and the implementation of negative controls further reassured that no systematic bias or residual confounding were observed, and the results were calibrated based on the empirical distributions of the negative controls [22]. Clinical interpretability of the findings is limited by the lack of stratification of prostatitis by severity (acute versus chronic) and whether prostatitis presents differently in SGLT2i users (e.g., bacterial versus nonbacterial). A broader definition of prostatitis was used in this study to address the specific Health Authority query; further stratification would have been limited by the use of ICD diagnosis codes. The analyses were conducted for 4 specific AHAs using data from 7 US databases and 1 Japanese database, with the majority of patients coming from the US (particularly from the Optum^®^ EHR, IQVIA^TM^ PharMetrics Plus, and IQVIA^TM^ Ambulatory EMR databases); therefore, the findings are not generalizable to other geographic regions and other AHAs. Regarding statistical methodology, although the Cox proportional hazard model that was used to estimate the HR for prostatitis within each database assumes proportionality of hazards, a quantitative evaluation of proportionality of hazards was not conducted. Finally, while there is an established relationship between lower urinary tract dysfunction or prostate enlargement and the development of prostatitis, these risk factors are beyond the scope of this study and would be important endpoints for future analyses.

This study, using a comparative cohort design with active comparators, found no evidence of a statistically significantly increased risk of prostatitis in adult male patients with type 2 diabetes mellitus who received canagliflozin compared with the other AHAs evaluated in this study, providing evidence for the lack of a safety issue for this hypothesized association. Future studies based on clinical datasets are needed to validate these findings. In addition, the potential for age-related effect modification (if any) confounding on the risk of prostatitis among SGLT2i users could be explored by future research.

## Supporting information

S1 Fig Covariate Balance Before and After Propensity Score Matching (IQVIA^TM^ Ambulatory EMR Database). EMR, electronic medical record.
(PDF)

S1 Table Baseline Demographics and Clinical Characteristics of the Canagliflozin Cohort. CCAE, Commercial Claims and Encounters; CDM, Clinformatics^®^ Data Mart; EHR, electronic health record; EMR, electronic medical record; JMDC, Japan medical data center; MDCD, multi-state Medicaid database; MDCR, Medicare supplemental; NA, not available; SD, standard deviation. ^a^ Select comorbidities and concomitant medications are those with a prevalence of at least 20% in any treatment cohort. Concomitant medications exclude those that were evaluated in this study (i.e., canagliflozin, dapagliflozin, empagliflozin, liraglutide, and sitagliptin).
(XLSX)

S2 Table Baseline Demographics and Clinical Characteristics of the Dapagliflozin Cohort. CCAE, Commercial Claims and Encounters; CDM, Clinformatics^®^ Data Mart; EHR, electronic health record; EMR, electronic medical record; JMDC, Japan medical data center; MDCD, multi-state Medicaid database; MDCR, Medicare supplemental; NA, not available; SD, standard deviation. ^a^ Select comorbidities and concomitant medications are those with a prevalence of at least 20% in any treatment cohort. Concomitant medications exclude those that were evaluated in this study (i.e., canagliflozin, dapagliflozin, empagliflozin, liraglutide, and sitagliptin).
(XLSX)

S3 Table Baseline Demographics and Clinical Characteristics of the Empagliflozin Cohort. CCAE, Commercial Claims and Encounters; CDM, Clinformatics^®^ Data Mart; EHR, electronic health record; EMR, electronic medical record; JMDC, Japan medical data center; MDCD, multi-state Medicaid database; MDCR, Medicare supplemental; NA, not available; SD, standard deviation. ^a^ Select comorbidities and concomitant medications are those with a prevalence of at least 20% in any treatment cohort. Concomitant medications exclude those that were evaluated in this study (i.e., canagliflozin, dapagliflozin, empagliflozin, liraglutide, and sitagliptin).
(XLSX)

S4 Table Baseline Demographics and Clinical Characteristics of the Liraglutide Cohort. CCAE, Commercial Claims and Encounters; CDM, Clinformatics^®^ Data Mart; EHR, electronic health record; EMR, electronic medical record; JMDC, Japan medical data center; MDCD, multi-state Medicaid database; MDCR, Medicare supplemental; NA, not available; SD, standard deviation. ^a^ Select comorbidities and concomitant medications are those with a prevalence of at least 20% in any treatment cohort. Concomitant medications exclude those that were evaluated in this study (i.e., canagliflozin, dapagliflozin, empagliflozin, liraglutide, and sitagliptin).
(XLSX)

S5 Table Baseline Demographics and Clinical Characteristics of the Sitagliptin Cohort. CCAE, Commercial Claims and Encounters; CDM, Clinformatics^®^ Data Mart; EHR, electronic health record; EMR, electronic medical record; JMDC, Japan medical data center; MDCD, multi-state Medicaid database; MDCR, Medicare supplemental; NA, not available; SD, standard deviation. ^a^ Select comorbidities and concomitant medications are those with a prevalence of at least 20% in any treatment cohort. Concomitant medications exclude those that were evaluated in this study (i.e., canagliflozin, dapagliflozin, empagliflozin, liraglutide, and sitagliptin).
(XLSX)

S6 Table Summary of Administrative Claims Databases Included in This Analysis. CCAE, commercial claims and encounters; CDM, Clinformatics^®^ Data Mart; EHR, electronic health record; EMR, electronic medical record; HIPAA, Health Insurance Portability and Accountability Act; MDC, medical data center; MDCD, multi-state Medicaid database; MDCR, Medicare supplemental; NLP, natural language processing.
(DOCX)

S7 Table International Classification of Diseases Codes Used to Define Prostatitis. CM, Clinical Modification; ICD, International Classification of Diseases.
(XLSX)

S8 Table Crude Incidence Rates and Event Counts of Prostatitis. CCAE, Commercial Claims and Encounters; CDM, Clinformatics® Data Mart; EHR, electronic health record; EMR, electronic medical record; IR, incidence rate; JMDC, Japan medical data center; MDCD, multi-state Medicaid database; MDCR, Medicare supplemental. Note: Data for the following comparisons are not presented because they failed pre-specified diagnostic testing: 1) canagliflozin vs. dapagliflozin – Optum CDM; 2) canagliflozin vs. liraglutide – JMDC.
(XLSX)
