# Supplementary material for: Risk of prostatitis in patients with type 2 diabetes mellitus: An observational retrospective cohort study of canagliflozin versus other antihyperglycemic agents using propensity score matching
Source: PLoS One. 2026 Feb 2;21(2):e0341745. doi: 10.1371/journal.pone.0341745 (PMC12863472; doi:10.1371/journal.pone.0341745)
Supplement: S1 Fig — EMR, electronic medical record. (PDF) [file pone.0341745.s001.pdf]

S1 Fig. Covariate Balance Before and After Propensity Score Matching (IQVIA™ Ambulatory EMR database)

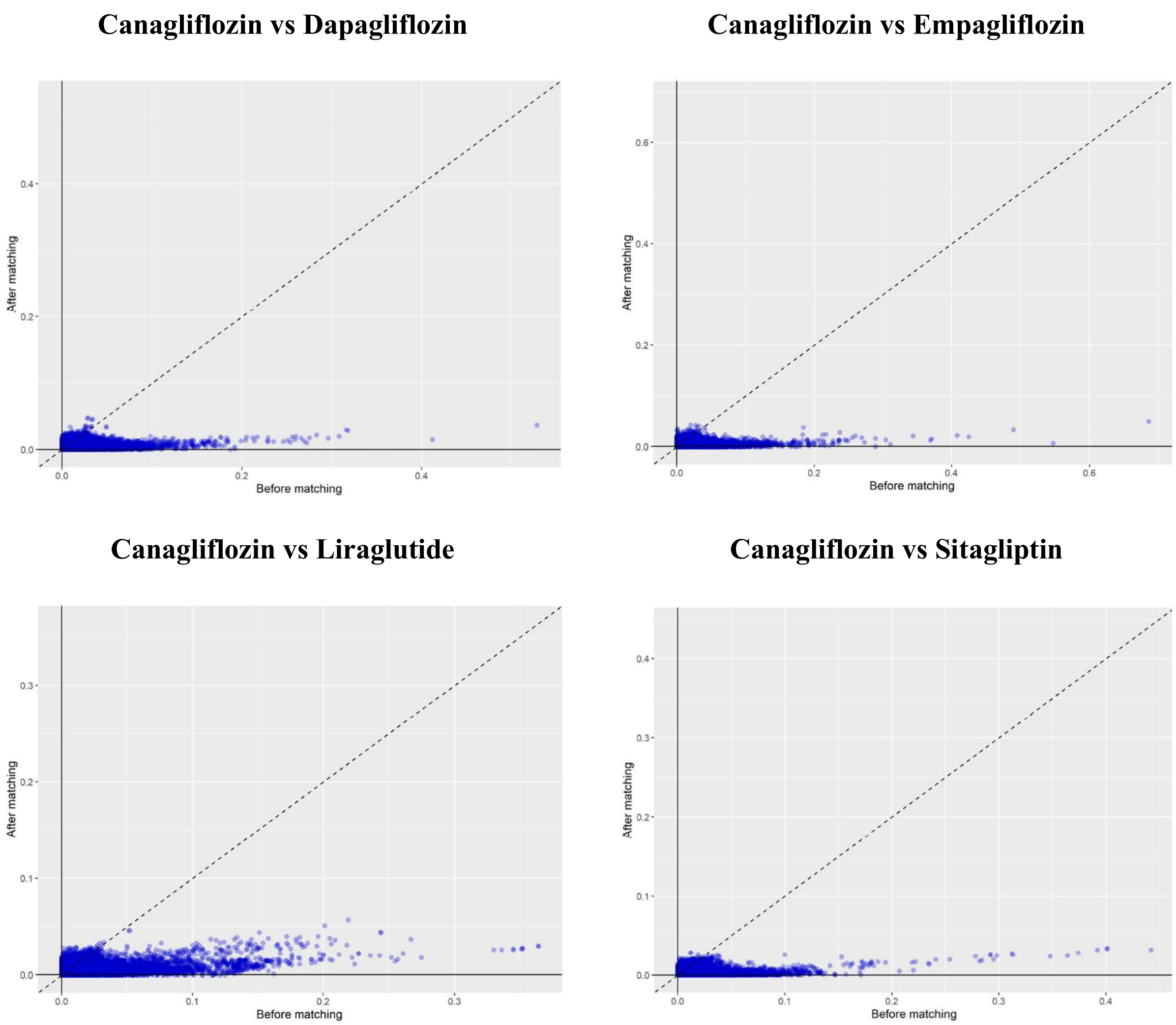

EMR, electronic medical record.
