# Supplementary material for: Risk of prostatitis in patients with type 2 diabetes mellitus: An observational retrospective cohort study of canagliflozin versus other antihyperglycemic agents using propensity score matching
Source: PLoS One. 2026 Feb 2;21(2):e0341745. doi: 10.1371/journal.pone.0341745 (PMC12863472; doi:10.1371/journal.pone.0341745)
Supplement: S6 Table — CCAE, commercial claims and encounters; CDM, Clinformatics® Data Mart; EHR, electronic health record; EMR, electronic medical record; HIPAA, Health Insurance Portability and Accountability Act; MDC, medical data center; MDCD, multi-state Medicaid database; MDCR, Medicare supplemental; NLP, natural language processing. (DOCX) [file pone.0341745.s007.docx]

**S6 Table. Summary of Administrative Claims Databases Included in This Analysis**

| Merative MarketScan^®^ CCAE | The Merative^®^ MarketScan^®^ CCAE includes health insurance claims across the continuum of care (eg, inpatient, outpatient, outpatient pharmacy, carve-out behavioral health care), as well as enrollment data from large employers and health plans across the United States who provide private health care coverage for employees, their spouses, and dependents. This administrative claims database includes a variety of fee-for-service, preferred provider organizations, and capitated health plans. This database encompasses primarily patients <65 years of age. |
| --- | --- |
| Merative MarketScan^®^ MDCR | The Merative^®^ MarketScan^®^ MDCR represents the health services of retirees in the United States with Medicare supplemental coverage through employer-sponsored plans. This database contains primarily fee-for-service plans and includes health insurance claims across the continuum of care (eg, inpatient, outpatient, and outpatient pharmacy). This database includes primarily patients >65 years of age. |
| Merative MarketScan^®^ MDCD | The Merative^®^ MarketScan^®^ MDCD reflects the health care service use of individuals covered by Medicaid programs in numerous geographically dispersed states. The database contains the pooled health care experience of Medicaid enrollees, covered under fee-for-service and managed care plans. It includes records of inpatient services, inpatient admissions, outpatient services, and prescription drug claims, as well as information on long-term care. Data on eligibility and service and provider type are also included. In addition to standard demographic variables, such as age and gender, the database includes variables, such as federal aid category (income based, disability, Temporary Assistance for Needy Families) and race. |
| Optum^®^ EHR | Optum^®^’s longitudinal EHR repository is derived from dozens of health care provider organizations in the United States that include >57 contributing sources and 111,000 sites of care. The data are certified as de-identified by an independent statistical expert following HIPAA statistical de-identification rules and managed according to Optum^®^ customer data use agreements. Clinical, claims, and other medical administrative data is obtained from both inpatient and ambulatory EHRs, practice management systems, and numerous other internal systems. Information is processed, normalized, and standardized across the continuum of care from both acute inpatient stays and outpatient visits. Optum^®^ data elements include demographics, medications prescribed and administered, immunizations, allergies, lab results (including microbiology), vital signs and other observable measurements, clinical and inpatient stay administrative data, and coded diagnoses and procedures. In addition, Optum^®^ uses NLP computing technology to transform critical facts from physician notes into usable datasets. The NLP data provides detailed information regarding signs and symptoms, family history, disease-related scores (ie, RAPID3 for rheumatoid arthritis, or CHADS_2_ for stroke risk), genetic testing, medication changes, and physician rationale behind prescribing decisions that might never be recorded in the EHR. |
| Optum^®^ CDM | Optum^®^’s CDM is derived from a database of administrative health claims for members of large commercial and Medicare Advantage health plans. The database includes data over a 14-year period (January 2007 through December 2021). CDM is statistically de-identified under the expert determination method consistent with HIPAA and managed according to Optum^®^ customer data use agreements. CDM administrative claims submitted for payment by providers and pharmacies are verified, adjudicated, and de-identified prior to inclusion. These data, including patient-level enrollment information, are derived from claims submitted for all medical and pharmacy health care services with information related to health care costs and resource utilization. The population is geographically diverse, spanning all 50 US states. |
| IQVIA™ Ambulatory EMR | The IQVIA™ Ambulatory EMR asset is comprised of patient records generated from face-to-face physician interactions since 2006 and are sourced from an “opt-in” provider research network. The aggregated database comprises records collected by >100,000 physicians from large practices and physician networks located in all 50 US states. Approximately 40% of the contributing physicians are primary care practitioners and the remaining are specialists. Key information collected includes patient demographics, vital signs, lab tests performed and results, allergies and vaccine details, diagnoses, prescription drugs prescribed and administered, procedures, and patient care episodes. |
| IQVIA™ PharMetrics Plus | The IQVIA™ PharMetrics Adjudicated Health Plan Claims Data (formerly PharMetrics Plus) is a US database comprised of fully adjudicated health plan claims data and enrollment information for commercial individuals. The information is comprised of >70 contributing health plans and self-insured employer groups throughout the United States over the last 5 years. This anonymous, patient-centric database includes all medical and pharmacy claims data (costs and descriptive services). Claims represent payments to providers for services rendered to covered health plan individuals. The data also includes patient-level enrollment that is a record of demographic variables including eligibility status (year of birth, gender, US Census region, eligibility by month). The enrollee population in the database is generally representative of the <65 years of age, commercially insured population with a subset of commercial Medicare and Medicaid in the United States with respect to both age and gender. |
| JMDC | The JMDC database consists of data from >250 health insurance associations covering workers aged <75 and their dependents. The proportion of patients who are <66 years old in the JMDC is approximately the same as the proportion in the whole nation. JMDC data includes data on membership status of the insured people and claims data provided by insurers under contract. Claims data are derived from monthly claims issued by clinics, hospitals, and community pharmacies. The size of JMDC population is about 10% of people in the whole nation. |

CCAE, commercial claims and encounters; CDM, Clinformatics^®^ Data Mart; EHR, electronic health record; EMR, electronic medical record; HIPAA, Health Insurance Portability and Accountability Act; MDC, medical data center; MDCD, multi-state Medicaid database; MDCR, Medicare supplemental; NLP, natural language processing.
